# Use of Superelastic Nitinol and Highly-Stretchable Latex to Develop a Tongue Prosthetic Assist Device and Facilitate Swallowing for Dysphagia Patients

**DOI:** 10.3390/ma12213555

**Published:** 2019-10-30

**Authors:** Mahdis Shayan, Neil Gildener-Leapman, Moataz Elsisy, Jack T. Hastings, Shinjae Kwon, Woon-Hong Yeo, Jee-Hong Kim, Puneeth Shridhar, Gabrielle Salazar, Youngjae Chun

**Affiliations:** 1 Department of Cardiothoracic Surgery, School of Medicine, Stanford University, Falk Building, Room CV-035, 870 Quarry Rd, Palo Alto, CA 94304, USA; mahdis.shayan@stanford.edu; 2 Department of Surgery, Albany Medical College, 43 New Scotland Ave., Albany, NY 1228, USA; gildenn2@mail.amc.edu; 3 Department of Industrial Engineering, University of Pittsburgh, 522 Benedum Hall, 3700 O’Hara Street, Pittsburgh, PA 15261, USA; mme41@pitt.edu; 4 Department of Bioengineering, University of Pittsburgh, 522 Benedum Hall, 3700 O’Hara Street, Pittsburgh, PA 15261, USA; jackhastings@pitt.edu (J.T.H.); PUS8@pitt.edu (P.S.); gfsalazar2000@yahoo.com (G.S.); 5 George W. Woodruff School of Mechanical Engineering, Institute for Electronics and Nanotechnology, College of Engineering, Georgia Institute of Technology, Atlanta, GA 30332, USA; skwon64@gatech.edu (S.K.); whyeo@gatech.edu (W.-H.Y.); 6 Wallace H. Coulter Department of Biomedical Engineering, Parker H. Petit Institute for Bioengineering and Biosciences, Institute for Materials, Neural Engineering Center, Institute for Robotics and Intelligent Machines, Georgia Institute of Technology, Atlanta, GA 30332, USA; 7 Department of Otolaryngology, School of Medicine, University of Pittsburgh, 3550 Terrace Street, Pittsburgh, PA 15261, USA; je104@pitt.edu; 8 McGowan Institute for Regenerative Medicine, University of Pittsburgh, Pittsburgh, PA 15219, USA

**Keywords:** dysphagia, swallowing, tongue, nitinol, superelastic, prosthesis

## Abstract

We introduce a new tongue prosthetic assist device (TPAD), which shows the first prosthetic application for potential treatment of swallowing difficulty in dysphagia patients. The native tongue has a number of complex movements that are not feasible to mimic using a single mechanical prosthetic device. In order to overcome this challenge, our device has three key features, including (1) a superelastic nitinol structure that transfers the force produced by the jaws during chewing towards the palate, (2) angled composite tubes for guiding the nitinol strips smoothly during the motion, and (3) highly stretchable thin polymeric membrane as a covering sheet in order to secure the food and fluids on top of the TPAD for easy swallowing. A set of mechanical experiments has optimized the size and angle of the guiding tubes for the TPAD. The low-profile TPAD was successfully placed in a cadaver model and its mobility effectively provided a simplistic mimic of the native tongue elevation function by applying vertical chewing motions. This is the first demonstration of a new oral device powered by the jaw motions in order to create a bulge in the middle of the mouth mimicking native tongue behavior.

## 1. Introduction

Millions of Americans suffer from swallowing disorders primarily due to dysphagia every year [1,2]. Dysphagia occurs when the patient cannot properly transfer the food from the mouth to the esophagus or from the esophagus to the stomach. It leads to negative health outcomes and reduces quality of life due to gastrostomy dependence, aspiration pneumonia, malnutrition, as well as loss of physical and social pleasure experienced through eating. 

Current treatment options for dysphagia include: postural strategies for head or body position, change in food bolus volume or viscosity, tonic muscle contraction including neuromuscular electrical stimulation (NMES), sensorial enhancement strategies, and gastrostomy tube alimentation and surgery [3]. A widely accepted rehabilitation approach is the Madison Oral Strengthening Therapeutic (MOST) system that provides pressure feedback for isometric tongue exercises [4]. Recently, people have studied methods to use human–computer interactions for feedback-loop rehabilitation [5,6]. However, when these therapies fail, there is no intervention available that could replace lost swallow functionality and address the decreased quality of life. 

Therefore, we introduced a novel non-invasive prosthetic device that, in part, mimicked the elevation function of the native tongue. While some static devices such as palatal drop prostheses can increase intraoral pressures, all other prevailing oral appliances for defect compensation and swallowing aids are static devices [7]. However, the potential benefit of a dynamic prosthetic device is that it provides additional room when loading a food bolus when the device is in its relaxed state. Conversely, when the device is deployed, pressures would gradually increase to propel the food bolus towards the pharynx. Our unique device would provide immediate mechanical strength to propel the food bolus into the pharyngeal phase of swallowing. The design of the tongue prosthetic assist device (TPAD) was based on exploiting the excellent and unique mechanical properties of nitinol (i.e., superelasticity and high fatigue resistance) [8]. The nitinol biomaterials were used to compensate for the pressure that the restricted tongue muscles cannot provide.

**Figure 1:**
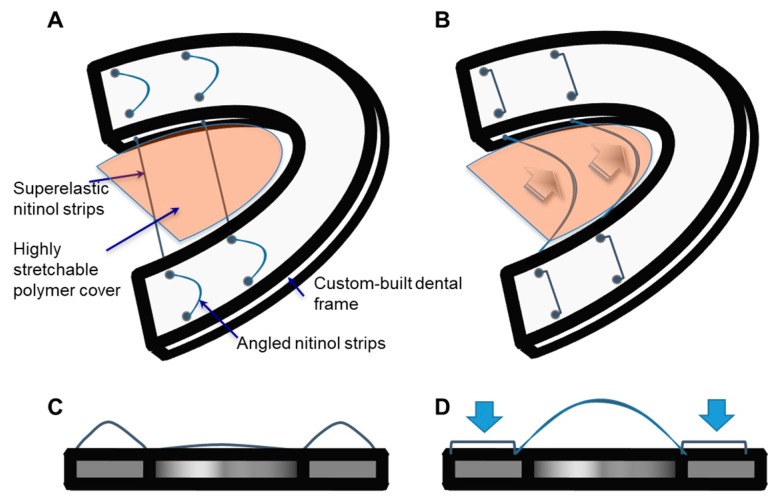
The schematic of the tongue prosthetic assist device (TPAD): dorsal–lateral view before applying the force (**A**) and after applying the force (**B**), front view before applying the force (**C**) and after applying the force (**D**).

In the present work, we have designed and manufactured a functional prototype, as shown schematically in Figure 1, through in vitro resistance evaluation, pressure measurement, and subsequently tested with human cadaver model. The design parameters used in developing TPAD were fully investigated and in vitro pressure measurement study results demonstrated the performance of the device. 

## 2. Materials and Methods 

### 2.1. Superelastic Nitinol and Acrylic Resin

Two materials were primarily used for the prototype construction, i.e., superelastic nitinol and denture acrylic resin. The superelastic nitinol biomaterials were used for the dynamic backbone in the tongue prosthetic assist device (TPAD). Mechanically drawn superelastic nitinol strips (width × thickness = 0.0240” × 0.0135”, Confluent Medical, CA) provided a stable motion for creating a middle bulge when the mouth was closed, then, the nitinol strips returned to their initial position with their own elastic property. Another important material used in TPAD was acrylic resin that contains denture acrylic resin (Opti-Cryl, US Dental DEPOT, FL) and instant tray mix acrylic resin (Lang Dental, IL). This acrylic resin material was used to fabricate a custom-made dental model for tests.

### 2.2. Fabrication Processes of a TPAD Prototype

A structural frame for embedding modular segments was fabricated using both a disposable impression tray (Bosworth Tray Aways^®^, Bosworth, IL, USA) and dental impression kit (Flexitime, Heraeus Kulzer GmbH, Hanau, Germany). First, the impression of the cadaver’s teeth was acquired by inserting the plastic tray containing putty material (i.e., dental impression kit) inside the cadaver’s mouth and pressing it around the teeth. Over time, the putty material hardened, and a stiff negative impression was formed. The resulting impression mold was filled with Opti-Cryl acrylic resin in order to prepare the dental impression stone model after hardening the acrylic resin. The final acrylic tray was prepared by placing the mixture of acrylic resin monomer and hardener on top of the stone mold, then, the low-profile dental tray was subsequently separated after the completion of in situ polymerization.

**Figure 2:**
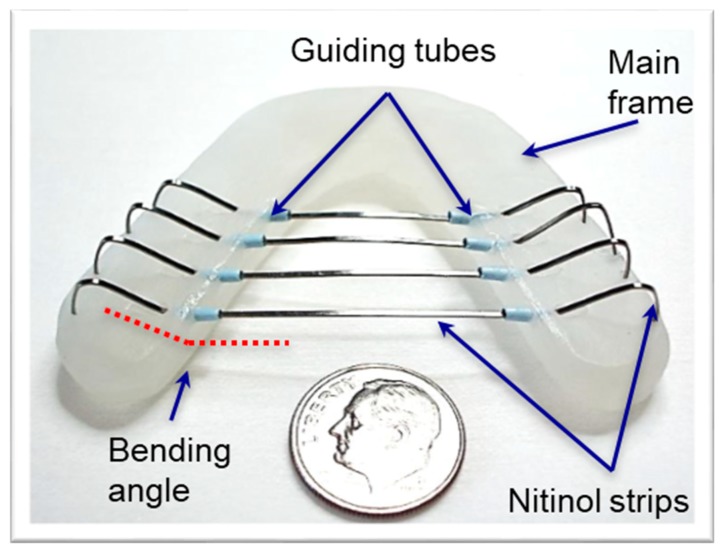
Information about TPAD, showing details of the components used in the prototype tongue indicating guiding tubes, bending angles, and device skeletal structure.

Once the dental frame was successfully prepared, multiple holes were creased via a precision drilling process with the designated angles. Then, composite tubes were bent and subsequently fixed on the frame. Multiple nitinol strips were pre-shaped using stress-induced deformation processes, then inserted through the pre-embedded composite tubes. Finally, a highly stretchable latex covering membrane was attached on the nitinol backbone. Figure 2 shows the prototype of TPAD, indicating the device components and sizes.

### 2.3. In Vitro Mechanical Property Measurements

#### 2.3.1. Resistance Measurement between Nitinol Strip and Guiding Tubes

The resistance that occurred during the sliding motion of nitinol strips in guiding tubes were measured using a mechanical test system (FLC-5E, Starrett, Athol, MA, USA) that pulled the nitinol strip through the guiding tubes to characterize resistance. A single superelastic nitinol strip was first placed in the guide tube. The load cell attached to this mechanical test system measured the exerted resistance during the pulling process. Three different bending angles of tubes, such as 90°, 120°, and 150°, were used to quantify the bending angle associated resistance. Both the length and diameter of the guiding tubes were also varied and used for the resistance studies. Three tube lengths were 7, 9, and 11 mm, and two tube diameters were 5Fr (ID: 1.67 mm) and 6Fr (ID: 2.0 mm) sizes, as shown in Table 1. With the various parameters used in the prototype, resistance was measured during the sliding motion of the superelastic nitinol strip in the curved guiding tubes.

#### 2.3.2. Tongue Pressure Measurements 

**Figure 3:**
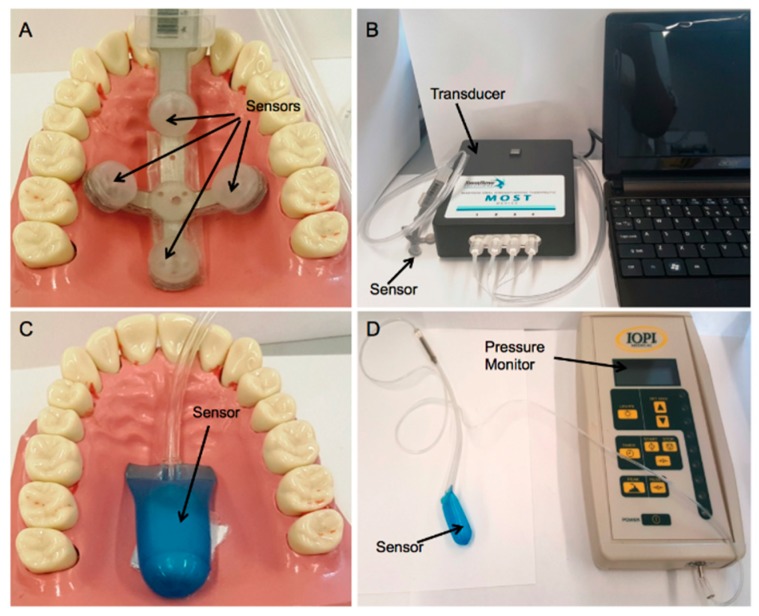
(**A**) MOST sensors placed in the palate of a mouth model, (**B**) MOST pressure measurement system, (**C**) IOPI sensor placed in the palate of a mouth model and, (**D**) IOPI pressure measurement system.

Two commercially available pressure measurement systems, the IOPI (Iowa Oral Performance Instrument, IOPI^®^ Model 2.3, Redmond, WA, USA) and MOST (Madison Oral Strengthening Therapeutic, Swallow solutions Model 1.5, Madison, WI, USA) were used to measure the pressure produced by the TPAD. Both systems have been already used in exercise therapy to assist patients with oral dysphagia, therefore, the measured pressure levels were helpful to refine the prototypes. The MOST pressure measurement system has four pressure sensors (Figure 3A) and a MOST laptop interface (Figure 3B). The IOPI system contains a balloon sensor (Figure 3C) that is connected to the monitor (Figure 3D).

### 2.4. In Vitro Cadaver Test

Under the approval of the Committee for Oversight of Research and Clinical Training Involving Decedents (CORID ID # 433) at the University of Pittsburgh, two human cadaver heads were used to evaluate the performance of the TPAD. Because it is important to hold the cadaver heads firmly in order to perform both X-ray imaging and pressure measurement, the cadaver head was fixated within a metal frame. The custom-made dental tray was prepared from the dental impressions of the cadaveric models. The X-ray imaging technique was used in order to evaluate the movement of the TPAD in the cadaver model. Both the front and lateral images of the TPAD were recorded while the mouth was open and closed. The pressures produced by the TPAD were measured and recorded using both IOPI and MOST systems by placing the sensors on the palate of the mouth in the cadavers. Before and after the use of the cadaver head models, they were stored in ethyl alcohol (Fisher Scientific, Pittsburgh PA, USA).

### 2.5. Statistical Analysis

The values of measured pressures were expressed as the mean value ± standard deviation (SD). Statistical analysis was performed for each experiment using one-way analysis of variance (ANOVA) test and Tukey’s honest significant difference (HSD) test by Minitab^®^ 16.1.0 (©2010 Minitab Inc. State College, PA, USA) and a *p*-value < 0.01 was considered statistically significant.

## 3. Results

### 3.1. Resistance in the Guiding Tubes

**Figure 4:**
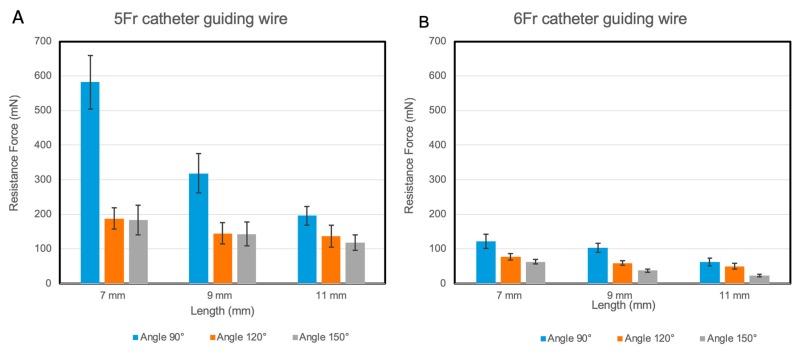
The relationship between the guiding tube length and the resistance during the nitinol wire movement in three different bending angles of 90°, 120° and 150°. (**A**) In a 5Fr catheter guiding tube and (**B**) in a 6Fr catheter guiding tube.

Figure 4A,B represent the relationship between the length of the guiding tubes and resistance force during the nitinol wire movement. In the 5Fr catheter (Figure 4A), the lengthening of catheter tubes from 9 mm up to 11 mm significantly decreased the resistance force since lengthening the catheters provided a smoother surface for sliding the nitinol wire. The level of this decrease was the most in smaller bending angles (i.e., 90° and 120° bending angle) compared to 150° bending angle. Lengthening the catheter tubes from 9 mm up to 11 mm in 120° and 150° bending angles did not significantly reduce the resistance force while in the 90° bending angle, this length change significantly decreased the resistance force. As for the 6Fr catheters shown in Figure 6B, the 6Fr catheters had a larger diameter and the nitinol wire slid in it with less friction; therefore, the resistance force in the 6Fr catheter was lower compared with it in a similar condition in the 5Fr catheter. In addition, lengthening the 6Fr catheter tubes slightly decreased the resistance force. The resistance force on average was higher in the 5Fr catheter compared to the 6Fr catheter (*p*-value < 0.01), the smaller diameter of the 5Fr catheter compared to the 6Fr catheter limits the freedom of the nitinol wire. Therefore, the lowest resistance force was observed in the 6Fr guiding tube with 150° bending angle (i.e., 22.9 ± 3.48 mN).

### 3.2. Prototype Fabrication

**Figure 5:**
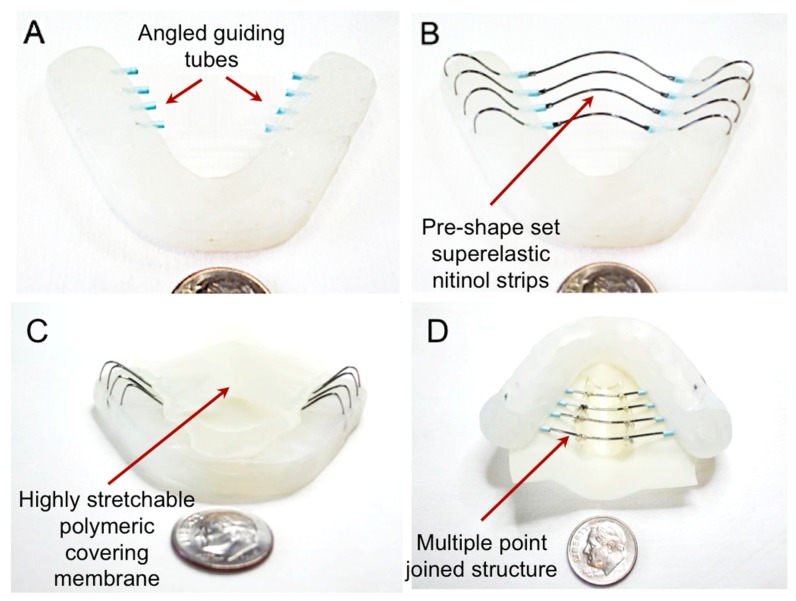
Fabrication steps of TPAD: (**A**) custom-built acrylic structural frame with catheter guiding tubes embedded into it, (**B**) nitinol strips placed in the mouth guard parallel to each other, (**C**) silicone layer covering on the parallel nitinol strips and, (**D**) the bottom view of the prototype.

Figure 5 shows the prototype development process of TPAD. The acryl dental model was constructed as described in Section 2.2. Guiding tubes after bending with angles of 90°, 120°, and 150° were successfully fixed in the frame (Figure 5A), and subsequently superelastic nitinol strips were placed after pre-shaping as shown in Figure 5B. Initial bulge was created based upon the anatomy of the cadaver model to effectively measure the exerted pressure levels. Figure 5C,D represent the fabricated TPAD prototype after covering the stretchable membrane via a mechanical suturing method. 

### 3.3. In Vitro Assessment of the Mechanical Performance of TPAD 

**Figure 6:**
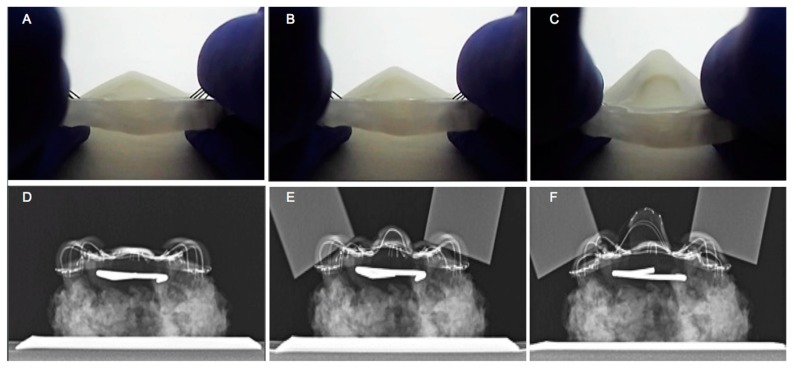
The representative TPAD in vitro performance: (**A**) before applying the force, (**B**) when it is half pressed, and (**C**) when it is totally pressed. X-ray images of the TPAD: (**D**) before applying the force, (**E**) after applying the force in the anterior position, and (**F**) in the posterior position.

The performance of the TPAD was qualitatively assessed by applying the mechanical force to the dental contact areas as shown in Figure 6A–C. Figure 6A shows the middle area configuration of the TPAD before applying any force to the dental contact areas. Once the force was applied to the dental contact areas, the middle area began to bulge (Figure 6B). Then, the dental contact areas were fully depressed, making the curved nitinol strips flat. The height of the central area increased by up to four times when the device was actuated (Figure 6C). Plain film X-rays were also used to evaluate the motion of the device by visualizing the nitinol structure. Figure 6D shows the nitinol structure before the application of an external force. Once the dental contact areas were pushed down using aluminum bars, the central area began to bulge upwards (Figure 6E), which represents the elastic deformation of the nitinol structures during jaw closure. Figure 6F represents the fully actuated nitinol structure when the dental contact areas were fully pushed down. More elastic deformation occurred during this motion, but the nitinol structure recovered its original geometry when the external force was removed (i.e., fully elastic deformation).

### 3.4. TPAD Performance Evaluation using Cadaveric Head Model

**Figure 7:**
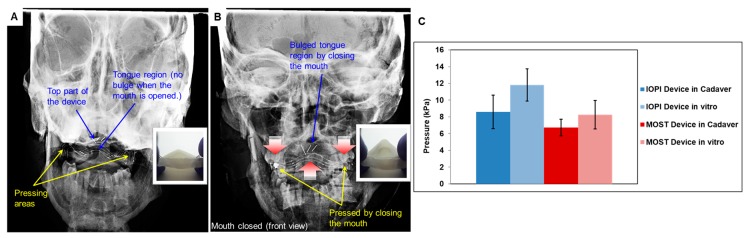
X-ray images of TPAD prototype placed in the cadaver mouth: front-view open mouth (**A**) and closed mouth (**B**). (**C**) Pressure measured by the Iowa Oral Performance Instrument (IOPI) and Madison Oral Strengthening Therapeutic (MOST) systems during the in vitro test.

Figure 7A,B show the representative front view X-ray images of the TPAD placed inside the cadaver mouth while the mouth was opened and closed, respectively. The device was successfully placed in the cadaver mouth due to its low-profile design as shown in Figure 7A. The dental contact areas were placed between mandibular and maxillary dentition. Three horizontally aligned wires on the top of the device were used as a maker of the top surface of the central area (the mechanical tongue). Once the mouth was closed, the dental contact areas were compressed and the three wires elevated centrally, the top surface made contact with the hard palate, increasing intraoral pressures, as the native tongue would typically do (Figure 7B). In the cadaver model, the force of the jaws was applied at a nearly 90 ° angle while in the in vitro performance test, the applied force was slightly inclined (~ 135 °) which resulted in a higher central bulge in the in vitro test.

The devices were also quantitatively evaluated by measuring the maximum exerted pressure levels during the biting motion. The pressure measured by MOST and IOPI did not always demonstrate similar pressure levels due to the different measurement mechanisms. The sensors of the MOST pressure measurement system are smaller and more sensitive compared to the one in the IOPI system and can record the maximum pressure with higher precision especially when the pressure is locally concentrated on a small area. The exerted tongue pressures by TPAD in the cadaver mouth were measured and compared with the in vitro laboratory pressure measurement results as shown in Figure 7. The pressures were decreased in the cadaver mouth by 25% in IOPI and 22.2% in MOST systems compared to the pressure produced during the in vitro laboratory tests, respectively.

## 4. Discussion

The tongue plays a significant role in swallowing, through preparing the bolus of food and pushing it posteriorly in order to transfer it from the oral to the pharyngeal phase of swallowing. Therefore, abnormal tongue performance can disturb swallowing whether at the oral preparatory or oral phase of swallowing [9,10,11]. The aim of the development of the TPAD was to augment the function of the impaired tongue by additional force on the food bolus to elevate it towards the palate and direct it posteriorly from the mouth to the pharynx. Our prototype uniquely demonstrated the concept of a simplistic prosthetic tongue through in vitro laboratory and cadaver testing. While these qualitative movements and exerted pressures were encouraging, our data did not prove clinical utility. The movements of the native tongue and pharynx are complex and coordinated, so the simplistic elevation movement of our device was only a partial mimic. To develop a device with potential clinical utility, several criteria were used in the design process. 

First, the biting force of normal people was analyzed in order to design a “biting force” based mechanical prosthetic tongue device. People typically move their jaw in multiple directions while eating. Mastication is complicated and thus the exerted biting force is not constant; biting force is an indicator of the masticatory system and is dependent on the gender, age, craniofacial morphology, type of the food, and the measurement techniques [12]. The human temporomandibular joint (TMJ) and muscles of mastication are capable of moving the mandible in anterior to posterior, lateral, and vertical displacement. The lateral and anterior–posterior chewing movements are more difficult to transfer into wire deformation. For this reason, only the vertical biting force was used to design TPAD. The exerted biting force in vertical direction must overcome the resistance force of the tongue prototypes. Gay et al. reported the maximum biting force in ten different subjects are between 25 to 400 N, which are significantly greater force values than the maximum resistance force (i.e., ~2 N) that was measured in our prosthetic tongue devices [13]. Therefore, the force that is exerted through chewing process is sufficient to operate the TPAD by overcoming the resistance force of the tongue prototypes [14,15]. 

Secondly, the pressure levels produced by TPAD were evaluated. IOPI medical device results show that the average strength of tongue (i.e., pushing force by tongue) for different ages is in the range between 50 and 75 kPa [10,16]. The tongue strengths of our prototype shown in this study ranged from 6 to 12 kPa depending on the design of prototype, such as the angle and geometry of guiding tubes, as well as the type of measurement systems (i.e., IOPI and MOST). The use of thick nitinol strips in more locations increased the mechanical tongue strength showing the maximum mechanical tongue pressure exerted by the TPAD. While the pressure exerted by the prosthetic device is an important design factor to be considered, the device should have a low-profile design that has a sufficient spring-back force to recover the nitinol strips original geometry when the biting force is removed. In addition, the value of the exerted pressures was further reduced in the cadaver compared to the in vitro pressure measurement test of the same prototype (Figure 7). The first reason was that the cadaver model was not sufficiently soft and easy to manipulate to apply for the vertical direction biting force to the device mimicking human jaw motion. The second reason was that the central area of the prototype (i.e., bulging region) was difficult to align with the placed pressure sensors during the measurement. The cadaver model was especially useful to demonstrate device fit in the human mouth. We qualitatively assessed the proper fit using in vitro X-ray visualization technique with the cadaver model. The patient specific TPAD was placed on the bottom teeth and moved well with the biting motion of the cadaver. The device successfully transferred the vertical direction force to the central area that created an upward bulge mimicking typical tongue motion during swallowing. While the device worked properly with cadavers, additional ceramic materials were added to stabilize the device on the mandible in these specimens, since the teeth were irregular, and some were missing. The device could have equally been used as a type of dynamic palatal augmentation prosthesis, affixed to the maxillary dentition. 

There are a number of clinical scenarios in which a TPAD-like device could be helpful to augment swallowing strength. These scenarios have all been cited in discussion of static palatal augmentation prostheses. In a patient with stroke-related dysphagia that maintains good airway protection, a TPAD could help augment the strength of swallow and treat delayed swallow. In anterior lateral sclerosis (ALS), the use of Palatal Augmentation Prosthesis (PAP) has been described [7]. In early bulbar onset ALS, the hypoglossal nerve may be affected first, with impaired oral control; subsequently laryngeal control and airway protection becomes impaired. There may be a window of opportunity for certain ALS patients to use a TPAD before the airway is at risk [17]. Perhaps a more ideal patient would be one with an isolated oral glossectomy defect. Those patients have been demonstrated to benefit from oral tongue static prosthetics, and many still maintain airway protection [18]. A more common patient might be an elderly sarcopenic patient with low muscular reserve after an acute illness. Within days into their hospitalization, they may develop tongue weakness and dysphagia [19]. Such patients may benefit from a TPAD type device during their rehabilitation to avoid prolonged hospitalization and potential gastrostomy.

A simplistic TPAD has been successfully designed, manufactured, and subsequently tested in vitro. The device mimicked the elevation of the native tongue that pushes the food bolus toward the palate and the back of mouth space. The unique superelastic property of nitinol wires used for the prototypes allows for a simple low-profile design. The device does not require any bulky component for transferring forces or recovering the original geometries. Even though our TPAD prototypes have not shown the equivalent strength of a native tongue, they were placed in the cadaver model with excellent fit. The results also demonstrate the functionality of the device showing the potential of the TPAD in clinical use. A limitation of this study was the small number of in vitro cadaver tests. The data presented in this manuscript can serve as a proof-of-concept for the use of new mechanical prosthetic tongue device fabricated using superelastic nitinol materials. More thorough in vitro studies using various designs are needed to demonstrate the optimal wire thickness and numbers, as well as the nitinol geometric design. Nevertheless, this study shows the first proof-of-concept stage toward a new tongue prosthetic device that utilizes superelastic nitinol. Future work will include the refinement of the prototype in order to increase the exerted pressure levels and device fatigue tests. Prospective prototypes will focus on anterior to posterior deployment of the pressure wave to encourage bolus transfer into the pharynx. In addition, soft food and viscous fluids will be used to evaluate swallowing capability with the device. Even with further refinements of the device design and functionality, one of the primary safety concerns that remains is to ensure controlled transfer of the food bolus from the oral cavity to the pharynx. We can envision that each prospective patient fitted for the TPAD would need instrumental evaluation by a speech pathologist. Patients with baseline aspiration and poor laryngeal protection would be contraindicated. Voluntary jaw contracture and coordination with a swallow would select against any patients with significantly altered mental status and low motivation for training on the TPAD.

## 5. Conclusions

In this work, we introduced a novel prosthetic device to increase an intraoral pressure during swallowing as the native tongue muscle generates a pressure to direct the food backward. The newly developed TPAD has been characterized in vitro using commercially available pressure measurement systems and human cadaver models. The experimental results have demonstrated the device feasibility for assisting in swallowing. Future studies will focus on a fatigue test, while examining the device performance in clinical conditions. In addition, we will study relevant swallowing behaviors with fluids and foods by adapting sequential motions of the tongue from anterior to posterior.

## Figures and Tables

**Table 1:** Parameters and values in resistance testing of guiding tubes, and bending angles for device skeletal structure.

Parameter	Values Tested
Tube Bending Angle	90°, 120° and 150°
Tube Length	7, 9 and 11 mm
Tube Diameter	5 and 6Fr
